# The human microbiome in clinical translation: from bench to bedside

**DOI:** 10.3389/fmicb.2025.1632435

**Published:** 2025-09-16

**Authors:** Jhommara Bautista, Carolina E. Echeverría, Iván Maldonado-Noboa, Sofía Ojeda-Mosquera, Camila Hidalgo-Tinoco, Andrés López-Cortés

**Affiliations:** ^1^Cancer Research Group (CRG), Faculty of Medicine, Universidad de Las Américas, Quito, Ecuador; ^2^Department of Medicine, New York University Grossman School of Medicine, New York, NY, United States; ^3^Jefe de Servicio de Oncología, Hospital Metropolitano de Quito, Quito, Ecuador

**Keywords:** human microbiome, clinical translation, precision medicine, biomarkers, microbiota transplantation

## Abstract

The human microbiome, once regarded as a passive passenger, is now recognized as a dynamic and essential determinant of human physiology, shaping immunity, metabolism, neurodevelopment, and therapeutic responsiveness across the lifespan. Advances in multi-omic technologies, experimental models, and computational approaches have revealed mechanistic insights into how microbial communities modulate host systems across diverse body sites, including the gut, skin, lungs, oral cavity, and reproductive tract. The clinical translation of this knowledge has begun to redefine early-life programming, cardiometabolic regulation, immune homeostasis, neuropsychiatric resilience, and cancer therapy response. Innovative strategies such as phage therapy, live biotherapeutics, precision nutrition, and microbiota transplantation illustrate the therapeutic potential of harnessing microbial functions to prevent or treat disease. In parallel, large-scale initiatives cataloging the microbiome of underexplored niches, such as the vagina and skin, are advancing health equity by broadening representation in microbial reference datasets. Yet significant challenges persist, including interindividual variability, incomplete functional annotation of microbial “dark matter,” and the absence of validated biomarkers. Addressing these gaps requires standardized methodologies, harmonized regulatory frameworks, and longitudinal studies across diverse populations. This review outlines the progress and remaining hurdles in translating microbiome science into clinical practice and concludes that the microbiome now stands at the forefront of a paradigm shift, transforming concepts of disease etiology, therapeutic design, and the future of individualized medicine.

## Introduction

The human microbiome, a complex and dynamic ecosystem of microorganisms, plays a fundamental role in regulating immunity, metabolism, and neuroendocrine signaling throughout life (Jyoti and Dey, 2025; Zheng et al., 2020; Farzi et al., 2018). Once considered passive bystanders, microbial communities are now recognized as active participants in maintaining health and contributing to disease pathogenesis via intricate crosstalk with host pathways across multiple organ systems (O’Riordan et al., 2025; Macpherson et al., 2023; Gilbert et al., 2025). Recent comprehensive reviews have underscored that the gut microbiota functions as both a guardian of host homeostasis and a driver of diverse pathologies, with implications spanning gastrointestinal, metabolic, immune, and neurological diseases (Chen F. et al., 2023; Li H. et al., 2022; Afzaal et al., 2022). Importantly, interindividual variability in microbiome composition, driven by diet, geography, host genetics, antibiotic exposure, and age, remains a key barrier to reproducibility and complicates the development of universally applicable diagnostic and therapeutic tools.

Advancements in high-throughput sequencing, multi-omics integration, and experimental modeling have revealed mechanistic insights into how the microbiome modulates host resilience or vulnerability to disease (Xu et al., 2024; Maifeld et al., 2021). These studies highlight the microbiome’s duality in health and disease, where shifts in taxonomic composition, functional gene profiles, and metabolite production can influence both protective and pathogenic outcomes (Afzaal et al., 2022). These discoveries are driving translational efforts across clinical disciplines, spurring development of targeted interventions such as probiotics, prebiotics, bacteriophage therapy, and microbiota transplantation (Federici et al., 2022; Huang et al., 2024). Yet, the therapeutic potential of the microbiome remains constrained by high interindividual variability and the absence of standardized microbial biomarkers (Gilbert et al., 2025).

Immune signaling emerges as a central conduit for microbiota-host interactions, with microbial metabolites and structural components influencing immune homeostasis across both mucosal and systemic compartments (Macpherson et al., 2023). Dysbiosis-related immune perturbations have been implicated in conditions ranging from inflammatory bowel disease (IBD) and diabetes to neuropsychiatric and cardiovascular disorders (Federici et al., 2022; O’Riordan et al., 2025; Maifeld et al., 2021). Particularly, pharmacological perspectives have emphasized the therapeutic relevance of microbiome modulation, proposing probiotics, engineered strains, and metabolite-based therapies as intervention-ready tools to restore immune balance and metabolic function (Chen F. et al., 2023; Li H. et al., 2022).

Recent advances have highlighted novel translational strategies, such as phage consortia targeting *Klebsiella pneumoniae* or intermittent fasting protocols, that reprogram the microbiome and attenuate inflammation in clinical contexts like IBD and metabolic syndrome (Federici et al., 2022; Maifeld et al., 2021). Concurrently, large-scale initiatives such as the Vaginal Microbial Genome Collection (VMGC) are shedding light on low-biomass ecosystems, expanding our understanding of microbial contributions to reproductive and systemic health (Huang et al., 2024).

Collectively, these insights reflect a paradigm shift in microbiome science: from descriptive associations to intervention-ready, mechanistically grounded models. The growing body of evidence positions the human microbiome at the center of precision medicine, where microbiota-informed diagnostics and therapeutics are increasingly recognized as integral to the prevention and treatment of complex diseases (Ma et al., 2024; Porcari et al., 2025; Afzaal et al., 2022). This review synthesizes key developments in the clinical translation of microbiome research, focusing on therapeutic applications, anatomical niche-specific insights, and the remaining challenges in integrating microbiome-based tools into precision medicine (Macpherson et al., 2023; Gilbert et al., 2025; Huang et al., 2024).

### Gastrointestinal tract microbiome

**Figure 1:**
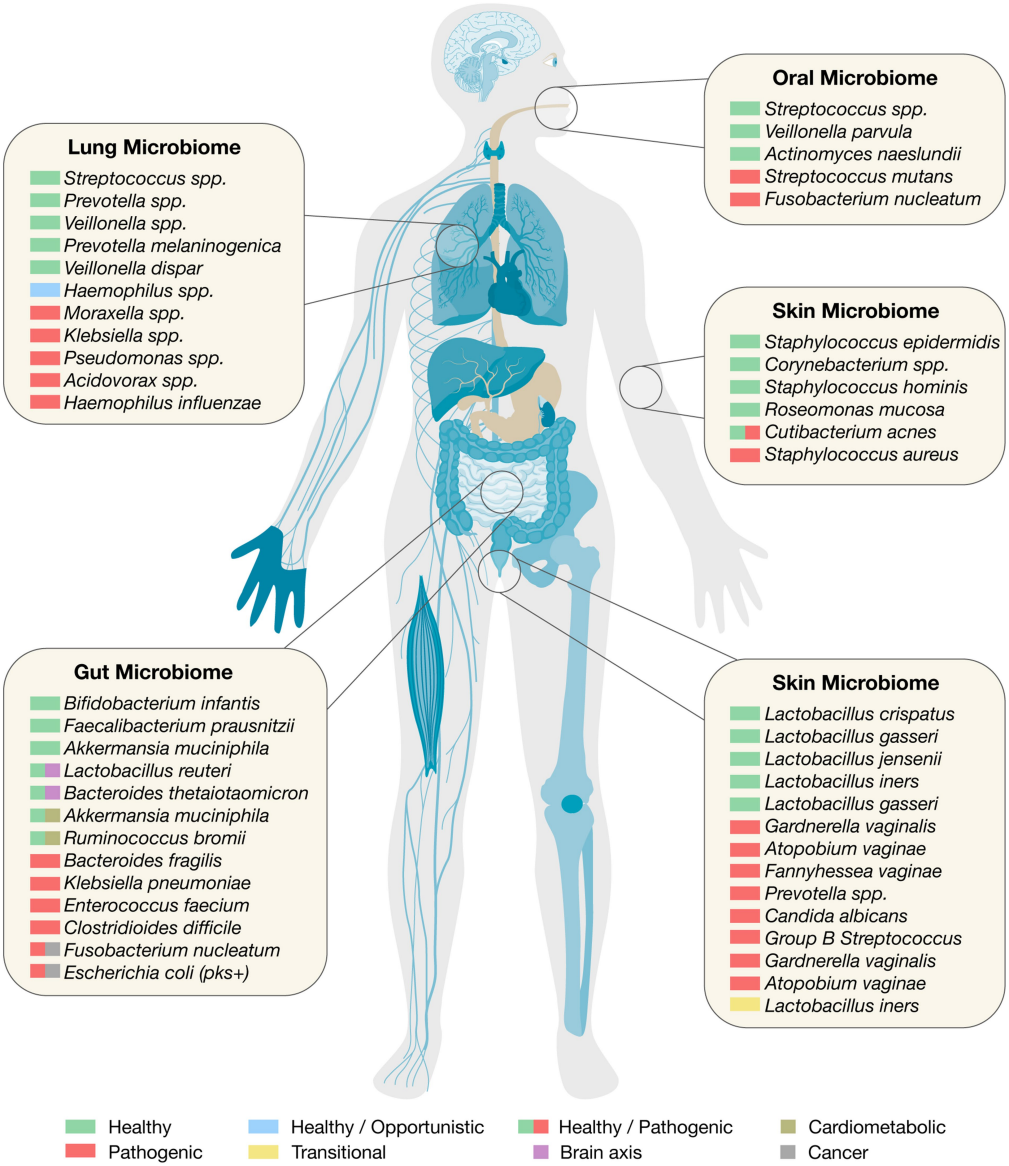
The human microbiome. Organ-specific healthy and pathogenic taxa relevant to clinical translation.

The gastrointestinal tract harbors one of the most complex and functionally diverse microbial ecosystems in the human body (Figure 1). From the moment of birth, this microbiome begins to shape immune development, metabolic programming, and mucosal integrity (Milani et al., 2017). However, the structure and function of the gut microbiota are not static; they evolve dynamically from infancy through adulthood and are shaped by environmental, dietary, and clinical exposures. Understanding the microbiome’s developmental trajectory, from the neonatal period to maturity, provides a critical foundation for targeted therapeutic strategies across the lifespan.

### The neonatal gut microbiome in early life programming

The neonatal period represents a foundational stage in human development during which the gut microbiome is seeded and begins to evolve, exerting long-lasting effects on host physiology, immunity, and metabolism. Colonization begins at birth and is largely dictated by maternal microbial transmission and environmental exposures. Vaginal delivery facilitates maternal transfer of *Lactobacillus*, *Prevotella*, and *Sneathia*, which colonize the neonate’s gastrointestinal tract (Dominguez-Bello et al., 2010). By contrast, cesarean delivery is associated with enrichment of skin-derived taxa such as *Staphylococcus* and *Corynebacterium*, reduced maternal transmission of *Bacteroides*, and delayed acquisition of commensals like *Bifidobacterium*, producing a distinct microbial profile from that of vaginally delivered infants (Dominguez-Bello et al., 2010; Shao et al., 2019; Reyman et al., 2019). This early divergence has been linked to increased risk of immune dysregulation and metabolic disorders, including obesity, asthma, and allergies, later in life (Yuan et al., 2016). Longitudinal studies have shown that while infancy is marked by rapid microbial succession, the gut microbiome generally reaches a stable, adult-like configuration by approximately 2–3 years of age (Yatsunenko et al., 2012; Stewart et al., 2018). This developmental milestone marks the establishment of the core microbiome, which provides functional resilience but remains modifiable by diet, antibiotic exposure, and geography throughout life (Tamburini et al., 2016).

Preclinical models have confirmed that these early microbial differences are not merely compositional but also functional. For instance, neonatal mice inoculated with vaginal microbiota from women dominated by *Lactobacillus crispatus* versus those with *Gardnerella vaginalis* and *Atopobium vaginae* show differential outcomes in metabolism, immune function, and neurodevelopment (Jašarević et al., 2021; Jašarević et al., 2018). These effects are modulated further by the maternal environment during gestation, particularly in cases of maternal obesity or vaginal dysbiosis, underscoring the interplay between prenatal and postnatal microbial exposures in determining offspring health trajectories (Márquez Ibarra et al., 2025).

Postnatal nutrition plays a critical role in shaping the early-life gut microbiome and immune development. Breastfeeding not only supplies essential nutrients but also delivers maternal microbes and bioactive compounds, notably human milk oligosaccharides (HMOs), which are pivotal in guiding microbial colonization (Kijner et al., 2022). HMOs serve as selective substrates for *Bifidobacterium infantis*, a key early colonizer that has co-evolved with the human host to dominate the infant gut niche. Colonization by *B. infantis* promotes immune homeostasis by suppressing pro-inflammatory Th2 and Th17 cytokines, enhancing IFN-*β* expression, and activating immunoregulatory pathways such as galectin-1 signaling (Henrick et al., 2021). Disruption of this delicate microbial succession can have lasting immunological consequences. A recent prospective birth cohort study showed that neonatal antibiotic exposure significantly impaired vaccine-induced antibody responses, an effect attributed to the depletion of beneficial *Bifidobacterium* species during critical windows of immune programming (Ryan et al., 2025).

While breastfeeding is a dominant driver of early microbial colonization, formula feeding and mixed feeding also exert significant effects on gut diversity and physiology. Formula-fed infants typically harbor lower abundances of *Bifidobacterium* and *Lactobacillus*, with increased colonization by *Clostridium* and *Enterobacteriaceae*, resulting in a more adult-like microbiota in early life (Penders et al., 2006; Stewart et al., 2018). This microbial divergence has been associated with altered SCFA production, heightened gut permeability, and increased risk of immune-mediated disorders (Ho et al., 2018). Mixed-fed infants often display intermediate microbial profiles, reflecting contributions from both breast milk and formula (Wang M. et al., 2025). Emerging strategies incorporating prebiotics, probiotics, or synthetic human milk oligosaccharides into infant formula show potential to partially restore the protective functions of breastfeeding, improving microbiota composition and supporting immune development (Puccio et al., 2017; Hedrick et al., 2021).

Beyond HMOs, breast milk also contains complement proteins that shape the gut microbial landscape through a C1-dependent, antibody-independent mechanism. These proteins selectively lyse specific gram-positive commensals, promoting the establishment of a microbial community that confers resistance against enteric pathogens (Pietrasanta et al., 2024). Experimental models have demonstrated that neonates deprived of complement-containing milk exhibit dysbiosis and heightened susceptibility to infections, supporting the immunomodulatory function of breast milk beyond passive immunity (Xu et al., 2024).

In vulnerable populations such as preterm infants, where microbiota development is often disrupted, clinical interventions have shown promising results. A large network meta-analysis concluded that prophylactic administration of multistrain probiotics, with or without prebiotics or lactoferrin, significantly reduces the incidence of severe necrotizing enterocolitis, sepsis, and feeding intolerance, while also shortening the time to full enteral feeding and hospital stays (Wang et al., 2023). These findings highlight the translational potential of microbiota-targeted strategies in neonatal care. Furthermore, targeted administration of *B. infantis* to undernourished infants has been shown to promote weight gain and reduce intestinal inflammation, offering a microbial solution to severe acute malnutrition in low-resource settings (Barratt et al., 2022).

Although cesarean-section births remain essential in many clinical situations, their impact on early microbial transmission has prompted interest in interventions such as vaginal microbiota transfer (Hourigan et al., 2022). A randomized controlled trial demonstrated that vaginal seeding in C-section-born neonates partially restores maternal microbial transmission and alters neonatal microbiota composition in both skin and stool (Mueller et al., 2023). These findings support the growing recognition that early microbial exposures are critical determinants of health, and that intentional modulation of the neonatal microbiome may serve as a novel therapeutic avenue. Overall, the neonatal gut microbiome is a dynamic and responsive ecosystem that plays a central role in early-life programming. Its development is governed by birth mode, maternal microbial reservoirs, breastfeeding, and nutritional interventions (Catassi et al., 2024).

### Maturation and plasticity of the adult gut microbiome

The adult gut microbiome represents a dynamic yet relatively stable ecosystem that plays a fundamental role in maintaining host homeostasis, influencing metabolic processes, modulating immune responses, and contributing to neuroendocrine signaling (Ma et al., 2024). In contrast to the neonatal microbiome, which undergoes rapid succession in early life, the adult gut microbiota preserves a stable core configuration while retaining flexibility to adapt to environmental factors, notably diet, antibiotics, and disease states (Tamburini et al., 2016; Aurora and Sanford, 2024). While host genetics exerts some influence on microbial composition, large-scale population analyses have demonstrated that environmental determinants, particularly diet and lifestyle, overwhelmingly outweigh genetic variation in shaping gut microbiota (Rothschild et al., 2018). Beyond genetics, cultural and geographic transitions can rapidly remodel microbial communities. A notable example is the US immigration study, which revealed that Southeast Asian immigrants experienced accelerated microbiome ‘westernization,’ characterized by reduced diversity and loss of fiber-degrading taxa, changes that paralleled increased metabolic risk (Vangay et al., 2018).

One of the most striking features of the adult gut microbiota is its metabolic versatility. The gut microbial community harbors immense genetic and enzymatic diversity, encoding ~150 times more genes than the human genome (Qin et al., 2010; Human Microbiome Project Consortium, 2012). This metabolic reservoir enables the biotransformation of dietary components, bile acids, xenobiotics, and host-derived molecules, with profound implications for health and disease (De Vos et al., 2022). For instance, secondary bile acids, produced through microbial transformation of host bile salts, exhibit anti-inflammatory properties and contribute to gut barrier integrity. Dysbiosis-induced depletion of these metabolites has been linked to intestinal inflammation, as seen in ulcerative colitis and pouchitis, where reduced abundance of *Ruminococcaceae* correlates with secondary bile acid deficiency and heightened inflammation (Sinha et al., 2020).

The adult microbiome also interfaces intricately with systemic metabolic regulation. In individuals with prediabetes, dietary interventions tailored to postprandial glycemic responses were shown to alter microbiota composition more significantly than traditional Mediterranean diets (Ben-Yacov et al., 2023). These changes, particularly increases in microbiota alpha diversity, were causally linked to improvements in hemoglobin A1c, lipid profiles, and weight control, underscoring the microbiome’s role as a mediator and modulator of cardiometabolic health (Napoli et al., 2024). Moreover, microbial metabolites such as short-chain fatty acids (SCFAs) and indole derivatives regulate pathways implicated in glucose metabolism, lipid balance, and inflammation, positioning the microbiome as both a target and effector of precision nutrition strategies (Zheng et al., 2022).

In adults, gut microbial composition is strongly associated with noncommunicable diseases beyond metabolic syndrome (West et al., 2015). Disorders such as IBD, colorectal cancer (CRC), rheumatoid arthritis, depression, and neurodegenerative diseases show consistent links with shifts in taxonomic composition, loss of microbial diversity, and altered functional gene profiles (Sun W. et al., 2024). Microbiome-wide association studies have become instrumental in identifying microbial signatures predictive of disease risk or therapeutic response. These studies highlight not only the importance of specific taxa but also functional genes, metabolites, and ecological interactions that underpin disease states (Gilbert et al., 2016, 2018). Importantly, single-cell and spatial tissue-omics approaches now link specific microbial and immune features to defined therapeutic outcomes in IBD: an IL-1–driven inflammatory fibroblast–neutrophil module marks multi-therapy non-response in deep ulcerative lesions (Friedrich et al., 2021); baseline enrichment of bile acid 7α/*β*-dehydroxylation (bai) gene–harboring *Clostridia* (e.g., the *Clostridium scindens* group) predicts response to anti-cytokine biologics (anti-TNF/ustekinumab) (Lee et al., 2021); higher baseline abundance of *Roseburia inulinivorans* and a *Burkholderiales* species is associated with clinical remission on vedolizumab (Ananthakrishnan et al., 2017); and pretreatment enrichment of Ki67^+^ memory CD4^+^ T cells identifies vedolizumab non-responders (Mennillo et al., 2024).

Antibiotic treatment is one of the most significant disruptors of the adult gut microbiome, often leading to increased vulnerability to infections (Ramirez et al., 2020). *Clostridioides difficile* infection (CDI) is a well-characterized example of this phenomenon, where antibiotic-induced depletion of key microbial taxa, particularly those involved in bile acid metabolism, creates a permissive environment for pathogenesis (Sulaiman et al., 2024). The loss of these taxa enriches conjugated bile acids that promote *C. difficile* spore germination while depleting secondary bile acids that normally inhibit its toxin activity. Restoring microbial bile-metabolizing functions has proven clinically effective in reducing CDI recurrence (Vinay et al., 2025). Interventions such as fecal microbiota transplantation (FMT) and next-generation live biotherapeutics like SER-109 reestablish microbial diversity and functionality, providing a mechanistic link between microbiota restoration and improved outcomes (Mullish and Allegretti, 2021; Feuerstadt et al., 2022). Complementary strategies, including probiotic co-administration during antibiotic therapy, have also demonstrated efficacy. A recent randomized controlled trial showed that multi-strain probiotics preserved microbial alpha diversity and significantly reduced the expansion of antibiotic resistance genes (John et al., 2024). Beyond CDI, emerging evidence highlights additional consequences of microbiome disruption. For instance, antibiotic-mediated depletion of *Clostridia* has been linked to sorbitol intolerance, a reversible phenotype corrected through targeted reintroduction of sorbitol-consuming bacterial strains. This murine study demonstrated that targeted probiotic reintroduction of sorbitol-consuming *Clostridia* corrected antibiotic-induced sorbitol intolerance, highlighting the potential of microbiota-based restoration strategies to mitigate antibiotic-induced metabolic dysfunction (Lee J. Y. et al., 2024).

Beyond the gastrointestinal tract, the adult gut microbiome exerts systemic effects through the gut–immune–brain axis. Microbial metabolites can influence neuroinflammation, glial cell function, and blood–brain barrier integrity (Loh et al., 2024). Studies have demonstrated that microbial composition correlates with neurological outcomes and that specific taxa modulate neuroactive compounds and neurotransmitter pathways (O’Riordan et al., 2025; Macpherson et al., 2023; Loh et al., 2024; Sinha et al., 2020). These findings are reshaping our understanding of the microbiota’s role in psychiatric and neurodegenerative conditions.

Altogether, the adult gut microbiome represents a highly adaptable and influential player in human health. Advances in multiomics, computational modeling, and clinical translation are paving the way for microbiome-based diagnostics and therapeutics (Rozera et al., 2025). However, challenges such as defining a “healthy” microbiome, accounting for interindividual variability, and establishing causal mechanisms remain. Nonetheless, the clinical potential of microbiome modulation, whether through diet, prebiotics, probiotics, live biotherapeutics, or microbiota-derived compounds, is becoming increasingly tangible (Ben-Yacov et al., 2023; Gilbert et al., 2025; Gilbert et al., 2018).

### The gut microbiota-brain axis in neurological health

The gut microbiota–brain axis is an intricate, bidirectional communication network that connects the gastrointestinal tract with the central nervous system (CNS), profoundly influencing brain development, behavior, mood, and cognition (Carabotti et al., 2015). This axis integrates neural, immune, endocrine, and metabolic pathways, many of which are shaped by microbial signals originating in the gut. Recent research has moved beyond descriptive correlations to uncover mechanistic insights into how microbial communities and their metabolites interact with the host’s nervous and immune systems to regulate neurodevelopmental and neurodegenerative processes (Zheng et al., 2020; O’Riordan et al., 2025).

Communication along the gut-brain axis occurs through multiple overlapping channels. Neural signaling is mediated predominantly via the vagus nerve, which transmits sensory information from the gut to the brain and modulates motor responses, immune tone, and gut physiology (Sun et al., 2023). The enteric nervous system (ENS), sometimes referred to as the “second brain,” operates autonomously but is tightly linked with both the gut microbiota and the CNS through neuroimmune and neuroendocrine pathways (Macpherson et al., 2023). Microbial metabolites such as SCFAs, neurotransmitters (e.g., serotonin, dopamine, GABA), and bile acid derivatives influence neuronal activity either locally within the ENS or systemically after crossing the blood–brain barrier (Agirman and Hsiao, 2021; Liu et al., 2022; Loh et al., 2024).

The immune system plays a central role in microbiota–brain communication. Gut microbes shape the development and function of microglia which are essential for synaptic pruning, neuronal maturation, and response to injury or disease (Loh et al., 2024). Disruption of gut microbial diversity can impair microglial maturation and trigger aberrant neuroinflammatory responses. These effects have been implicated in multiple CNS disorders, including depression, autism spectrum disorders (ASDs), and neurodegenerative diseases such as Alzheimer’s and Parkinson’s (Loh et al., 2024; O’Riordan et al., 2025; Liu et al., 2022). Notably, metabolites such as SCFAs and tryptophan metabolites regulate cytokine production, modulate the permeability of both the gut and blood–brain barriers, and influence the activation of peripheral immune cells that can traffic into the CNS under pathological conditions (O’Riordan et al., 2025; Sun X. et al., 2024).

In neurodegeneration, gut dysbiosis has been shown to contribute to disease pathogenesis. For example, alterations in microbial composition and metabolic output precede cognitive decline in Alzheimer’s disease models (Krishaa et al., 2023). Multi-omics approaches have identified key microbial metabolites that interact with orphan G-protein-coupled receptors (GPCRs) in the brain, regulating neuroinflammatory and neurodegenerative cascades (Qiu et al., 2024). Agmatine and phenethylamine, two gut-derived metabolites, were found to reduce tau hyperphosphorylation in iPSC-derived neurons from Alzheimer’s patients, offering proof-of-concept for microbiome-targeted interventions (Qiu et al., 2024).

Additionally, enteroendocrine cells in the gut epithelium act as sensors of microbial and nutritional cues, releasing gut hormones such as GLP-1 and PYY that signal to the brain via vagal afferents and modulate satiety, stress responses, and metabolic regulation (Barton et al., 2023). These mechanisms are particularly relevant in the context of obesity and eating disorders, where maladaptive gut-brain signaling contributes to disrupted energy homeostasis (Gruber et al., 2025).

Experimental evidence from germ-free and antibiotic-treated animal models has consistently demonstrated that the absence or alteration of the gut microbiota impacts neurogenesis, synaptic plasticity, and emotional behavior (Delgado-Ocaña and Cuesta, 2024; Luczynski et al., 2016). Transplantation of microbiota from individuals with depression into healthy rodents recapitulates depressive-like behaviors, highlighting the potential for microbial manipulation to alter brain function (Gheorghe et al., 2022). Beyond animal work, human evidence also supports a role for microbiota-targeted interventions in mood disorders. A systematic review of clinical FMT studies reported significant reductions in depressive and anxiety symptoms across multiple cohorts, underscoring translational relevance of these findings (Chinna Meyyappan et al., 2020). Importantly, longitudinal interventional studies in ASD have shown that FMT can significantly and durably reduce core ASD symptoms. In children with ASD and gastrointestinal comorbidities, open-label “microbiota transfer therapy” improved both gastrointestinal and behavioral outcomes, with benefits persisting at 2-year follow-up (Kang et al., 2017, 2019). More recently, an oral lyophilized FMT trial in children demonstrated sustained improvements in Autism Behavior Checklist and Childhood Autism Rating Scale scores, alongside enhanced sleep quality (Li Y. et al., 2024). Early adult and adolescent trials are now underway, reflecting growing interest in microbiome-targeted interventions for ASD.

The gut microbiota–brain axis constitutes a dynamic interface through which microbial communities influence CNS structure and function. Advances in mechanistic understanding, encompassing microbial metabolites, immune signaling, neurodevelopment, and neurotransmitter regulation, have opened new avenues for therapeutic strategies targeting the microbiome in neurodevelopmental, neuropsychiatric, and neurodegenerative disorders (Gheorghe et al., 2022; Jacobson et al., 2021; Macpherson et al., 2023; Liu et al., 2022). Lastly, early human trials suggest modest neuromodulatory potential of microbiota-based interventions. A recent double-blind, placebo-controlled study reported that a multi-strain probiotic supplement modestly improved subjective mood perception in healthy adults, although it did not significantly affect clinical depression scores, indicating subtle yet measurable effects on brain function (Johnson and Steenbergen, 2025).

### Microbial influences on cardiometabolic disorders

The gut microbiome has emerged as a central regulator of cardiometabolic health, exerting influence through complex interactions with host metabolism, immunity, and endocrine signaling (Tang et al., 2017). Dysbiosis, or the imbalance of microbial communities, has been strongly associated with key features of metabolic syndrome, including obesity, insulin resistance, dyslipidemia, and hypertension (Hassan et al., 2024).

Gut microbial metabolites are major mediators of these effects. SCFAs such as acetate, propionate, and butyrate, are produced through microbial fermentation of dietary fiber, enhance insulin sensitivity, regulate appetite, and support gut barrier integrity (Nogal et al., 2021). Conversely, metabolites like trimethylamine-N-oxide (TMAO), derived from microbial metabolism of choline and carnitine, have been implicated in the promotion of atherosclerosis and cardiovascular disease risk (Cao et al., 2022). Advances in systems biology have enabled deeper insights into these associations. Integrative analyses combining metagenomics, metabolomics, and clinical phenotyping have revealed that interindividual variation in microbial metabolite profiles correlates with diverse cardiometabolic traits, highlighting potential targets for intervention (Yang S. Y. et al., 2025; Sabih Ur Rehman et al., 2025). Complementing these findings, metaproteomic profiling has identified specific microbial proteins linked to SCFA biosynthesis and inflammatory pathways that are predictive of cardiovascular risk, opening avenues for non-invasive biomarker development (Yang C. et al., 2025). In addition to SCFAs and TMAO, emerging research has uncovered a novel microbial pathway involving the conjugation of amino acids to bile acids. This functional mechanism exerts immunomodulatory effects and further expands the known repertoire of host–microbiota interactions relevant to metabolic and inflammatory regulation (Lin et al., 2025).

Diet is a potent modulator of the gut microbiome and its metabolic output. In a randomized trial, supplementation with resistant starch for 8 weeks significantly improved insulin sensitivity and promoted weight loss in overweight individuals (Li H. et al., 2024). These benefits were attributed to enrichment of beneficial taxa such as *Bifidobacterium adolescentis*, modulation of bile acid metabolism, and attenuation of intestinal inflammation. Similarly, dietary fiber interventions have been shown to enhance microbial diversity and shift metabolic profiles toward anti-inflammatory phenotypes, underscoring the importance of microbial fermentation products in host metabolic regulation (Gilbert et al., 2025). Beyond fiber, caloric restriction and intermittent fasting also reshape the microbiota. A study in patients with metabolic syndrome demonstrated that a five-day fasting protocol, followed by a DASH-style refeeding regimen, resulted in sustained reductions in systolic blood pressure, BMI, and medication use (Maifeld et al., 2021). These effects were linked to shifts in microbial taxa capable of SCFA production and modulation of T-cell immune subsets, including Th17 and regulatory T cells. In addition, fermented dietary components offer another avenue for microbiota-targeted interventions. A recent randomized trial demonstrated that daily kombucha consumption enriched SCFA-producing gut microbes and led to modest reductions in systemic inflammation, supporting the potential of fermented beverages as adjunct therapies for cardiometabolic health (Ecklu-Mensah et al., 2024).

The microbiota’s contribution to weight regulation extends beyond acute effects. Microbiome composition and functional profiles at baseline have been found to predict individual responses to weight loss interventions, independent of BMI and diet (Diener et al., 2021). Functional traits such as bacterial replication rates and carbohydrate degradation pathways were associated with successful weight loss, highlighting the role of microbial ecology in shaping metabolic resilience. However, diet-induced microbiome changes are not always durable. Post-dieting weight regain, often seen in yo-yo dieting, has been mechanistically linked to persistent alterations in microbial composition that reduce energy expenditure and increase susceptibility to future weight gain. In murine models, this phenotype was transmissible via fecal microbiota transfer and was partially reversible by post-biotic interventions such as flavonoid supplementation (Thaiss et al., 2016). Recent human and animal evidence further implicates *Bacteroides vulgatus* and its metabolite pantothenate in dietary sugar preference and glucose homeostasis. Mechanistically, pantothenate-driven activation of free fatty acid receptor 4 (FFAR4) enhances GLP-1 secretion, thereby influencing satiety and sweet taste preference (Zhang et al., 2025). These findings extend the microbiome’s role from passive modulation of energy balance to active regulation of dietary behavior through gut–endocrine signaling.

Pharmacological and microbial therapies are being explored to restore metabolic balance. In patients with type 1 diabetes, adjuvant supplementation with probiotic strains such as *Lactobacillus salivarius*, *L. johnsonii*, and *Bifidobacterium animalis* resulted in decreased HbA1c levels and inflammatory cytokines, providing clinical support for microbiota modulation as a complementary strategy in glycemic control (Wang C. H. et al., 2022). Moreover, artificial sweeteners, widely used in attempts to reduce caloric intake, have paradoxically been shown to impair glucose metabolism by inducing dysbiosis. This highlights the need for careful evaluation of microbiome-related consequences of dietary interventions (Suez et al., 2014).

Finally, weight loss itself has been shown to remodel the metabolomic profile in type 2 diabetes. In the DiRECT trial, remission of diabetes was accompanied by favorable shifts in lipids, amino acids, and other metabolites, many of which were influenced by microbial metabolism (Corbin et al., 2024). These findings reinforce the concept that the microbiome is both a mediator and a marker of metabolic improvement. The gut microbiome plays a pivotal role in cardiometabolic health, with its composition, functional potential, and metabolic output all contributing to disease risk and therapeutic outcomes (Tang et al., 2017). Microbiome-informed interventions, whether dietary, probiotic, or pharmacologic, represent a promising frontier in personalized medicine for metabolic diseases (Gilbert et al., 2025; Cao et al., 2022; Li H. et al., 2024; Diener et al., 2021).

### Intersections between cancer, inflammation, and the gut microbiome

The relationship between cancer, inflammation, and the gut microbiome is multifaceted, reflecting a complex interplay between microbial communities, host immunity, and oncogenic processes (Lei et al., 2025). Accumulating evidence indicates that gut dysbiosis, characterized by loss of beneficial taxa and expansion of pro-inflammatory or genotoxic microbes, can both contribute to carcinogenesis and modulate the efficacy of cancer therapies (He et al., 2025).

Chronic inflammation is a known driver of tumorigenesis, and the gut microbiome plays a critical role in shaping inflammatory responses (Li Z. et al., 2024; Hanahan, 2022). Pathobionts such as *Klebsiella pneumoniae*, *Enterococcus faecium*, and *Bacteroides fragilis* have been identified in patients with IBD and CRC, and their expansion is often associated with disease exacerbation and immune dysregulation (Chow et al., 2011; Chandra et al., 2021). In experimental models, colonization with clinical IBD-derived *K. pneumoniae* strains induces intestinal inflammation, while targeted phage therapy directed at these strains has shown promise in reducing disease severity without disrupting commensal populations (Federici et al., 2022).

In cancer, the immunomodulatory capacity of the gut microbiota is particularly relevant for patients receiving immune checkpoint blockade (ICB) (Kang et al., 2024; Lei et al., 2025). Studies have demonstrated that specific bacterial taxa, including *Akkermansia muciniphila*, *Bifidobacterium pseudocatenulatum*, and *Faecalibacterium prausnitzii*, are enriched in responders to ICB and are associated with improved outcomes across multiple tumor types (Derosa et al., 2022; Lei et al., 2025). These microbes appear to enhance antigen presentation, T-cell activation, and tumor infiltration by CD8 + T cells (Li X. et al., 2022; Lee et al., 2022; Björk et al., 2024). Seminal murine studies first established causality for these interactions. Sivan et al. demonstrated that commensal *Bifidobacterium* promoted antitumor immunity and synergized with PD-L1 blockade (Sivan et al., 2015), while Vétizou et al. showed that CTLA-4 blockade required the presence of gut microbiota, with *Bacteroides fragilis* mediating therapeutic efficacy. These foundational discoveries laid the groundwork for subsequent clinical translation (Vétizou et al., 2015). Advancements in spatial omics technologies have further illuminated these microbiota–immune interactions at the tissue level. The MicroCart platform, for instance, enables high-resolution spatial profiling of microbial niches within inflamed colonic tissues, uncovering localized signatures of immune suppression that may facilitate tumor progression (Zhu et al., 2025).

FMT has emerged as a novel strategy to overcome ICB resistance. In patients with metastatic melanoma who failed prior PD-1 blockade, FMT from long-term responders restored responsiveness in a subset of patients. These clinical responses were accompanied by shifts in immune cell infiltration and tumor microenvironment reprogramming, supporting a causal role for the microbiome in modulating therapeutic outcomes (Baruch et al., 2021; Davar et al., 2021). Longitudinal profiling of these patients revealed that durable responders exhibited distinct microbial trajectories throughout treatment, marked by the stable or increasing abundance of immunoregulatory taxa, further supporting the link between microbiome dynamics and therapeutic outcomes (Björk et al., 2024). Building on these findings, synthetic biology approaches are being developed to engineer microbial therapeutics. For instance, genetically modified strains of *E. coli* Nissle 1917 have been designed to selectively colonize colorectal tumors and deliver immunomodulatory proteins directly within the tumor microenvironment, offering a novel means of enhancing antitumor immunity (Gurbatri et al., 2024).

Diet and lifestyle also influence this axis. Adherence to a Mediterranean diet, rich in fiber, polyphenols, and omega-3 fatty acids, has been associated with improved responses to ICB in melanoma patients (Bolte et al., 2023). This dietary pattern fosters a microbiome composition favorable for immunomodulation and may reduce immune-related adverse events. Similarly, dietary fiber and probiotic intake have been linked to improved ICB outcomes, reinforcing the notion that microbiota-targeted nutritional strategies can potentiate cancer therapy (Spencer et al., 2021). The mechanistic underpinnings of these associations involve microbial metabolites such as SCFAs, secondary bile acids, and tryptophan derivatives, which can influence immune cell function, epithelial barrier integrity, and systemic inflammation. For instance, SCFAs promote regulatory T cell expansion and mucosal homeostasis, while depletion of these metabolites in dysbiotic states exacerbates tumor-promoting inflammation (Li X. et al., 2022; Gilbert et al., 2025).

Collectively, these insights underscore the dual role of the gut microbiome as both a driver of cancer-related inflammation and a modifiable determinant of immunotherapy response (Xie et al., 2025). Integrating microbiome diagnostics and interventions such as FMT, probiotics, diet modification, and phage therapy into oncology could offer new avenues for enhancing treatment efficacy and reducing toxicity (Wang L. et al., 2025; Rivera-Orellana et al., 2025). However, challenges remain in standardizing microbial signatures, ensuring safety in immunocompromised patients, and understanding interindividual variability in microbiome-mediated responses (Gilbert et al., 2025; Li X. et al., 2022; Abdelsalam et al., 2023).

### Oral microbiome

The oral microbiome is a highly complex and dynamic microbial ecosystem that plays critical roles in both oral and systemic health (Figure 1). It ranks as the second most diverse microbial community in the human body after the gut, comprising over 700 species of bacteria as well as fungi, viruses, and protozoa (Peng et al., 2022). These microorganisms colonize distinct niches in the oral cavity, including the tongue, teeth, gingival crevice, hard and soft palates, and tonsillar tissues, each of which provides unique environmental conditions for microbial growth and interaction (Deo and Deshmukh, 2019; Mark Welch et al., 2020).

This microbial community is not randomly distributed. Instead, it is spatially structured into micron-scale biofilms shaped by host–microbe interactions, saliva flow, and intermicrobial competition. These biofilms form stratified architectures on oral surfaces, with taxa displaying habitat-specific patterns of colonization that enable complex polymicrobial cooperation or antagonism (Mark Welch et al., 2020; Hajishengallis et al., 2023). For example, *Streptococcus mutans*, a keystone pathogen in dental caries, establishes acidogenic and aciduric niches that promote enamel demineralization, while *Fusobacterium nucleatum* functions as a bridging organism that physically links early colonizers such as *Streptococcus sanguinis* with late colonizers including *Porphyromonas gingivalis*, a major periodontal pathogen (Hajishengallis et al., 2023; Lamont et al., 2018; Sedghi et al., 2021).

Periodontal diseases such as gingivitis and periodontitis are classic examples of how dysbiosis in the oral microbiome can drive chronic inflammation and tissue destruction (Hou et al., 2022). In a controlled experimental gingivitis model, localized inflammation was shown to propagate molecular changes in distant, clinically healthy oral tissues, highlighting how microbially induced inflammation can exert systemic effects even within the oral cavity (Kerns et al., 2023). These inflammatory responses vary among individuals and have been classified into distinct inflammatory responder types, underscoring the heterogeneity in host–microbe interactions.

Beyond oral health, the oral microbiome has systemic implications. Evidence increasingly links oral dysbiosis with diseases such as diabetes, cardiovascular disease, adverse pregnancy outcomes, and neurodegenerative conditions (Hajishengallis et al., 2023; Gao et al., 2018). Oral bacteria can translocate into the bloodstream, influence immune responses, and alter the inflammatory landscape of distal organs. For instance, *Fusobacterium nucleatum*, often enriched in periodontitis, has also been implicated in CRC (Hajishengallis et al., 2023; Peng et al., 2022). Host genetic factors also influence oral microbiome composition. Twin studies have shown that monozygotic twins share more similar oral microbial profiles than dizygotic twins, and that heritable bacteria tend to diminish with age and increased sugar intake. However, cariogenic species appear to be more environmentally driven, reflecting lifestyle, dietary habits, and hygiene practices (Gomez et al., 2017). Furthermore, lifestyle factors such as smoking and e-cigarette use significantly alter the oral microbiome (Chattopadhyay et al., 2024; Yang et al., 2023). E-cigarette users, despite appearing clinically healthy, exhibit oral microbial profiles similar to those found in severe periodontitis. These include higher representation of pathogenic species and enhanced pro-inflammatory signaling, indicating that such exposures may act as chronic perturbations that destabilize oral microbial ecosystems (Ganesan et al., 2020).

Advances in next-generation sequencing and metagenomics have facilitated deeper characterization of these microbial communities and their functions. The Human Oral Microbiome Database (HOMD) continues to serve as a central resource, cataloging species, genomic data, and associated phenotypes (Gao et al., 2018). Large-scale population studies have also revealed that the oral microbiome is shaped by age, health status, and social factors such as cohabitation or even classmate interactions, emphasizing the need to contextualize microbiome data within broader biological and sociocultural frameworks (Willis et al., 2022). Lastly, these insights underscore the oral microbiome’s relevance not only as a sentinel for oral diseases but also as a potential diagnostic and therapeutic target for systemic conditions. Efforts to modulate the oral microbiome, through improved hygiene, targeted antimicrobials, prebiotics, probiotics, or microbiota-informed precision interventions, offer promising avenues for maintaining health and preventing diseases (Hajishengallis et al., 2023; Gilbert et al., 2025).

### Lung microbiome

The lung microbiome, once believed to be negligible due to the presumed sterility of the lower respiratory tract, is now recognized as a critical modulator of pulmonary health and disease (Natalini et al., 2023; Figure 1). This ecosystem consists of a diverse array of microorganisms that colonize both the upper and lower airways. In healthy individuals, the lung microbiome maintains a delicate equilibrium, influenced by microbial immigration from the oral cavity and upper airways, as well as by clearance mechanisms such as mucociliary transport, alveolar macrophages, and surfactant activity (Belizário et al., 2023; Natalini et al., 2023; Li R. et al., 2024).

The microbial biomass in the lungs is low compared to the gut but exhibits a dynamic composition, shaped by constant microbial influx and selective clearance. Dominant taxa in healthy lungs typically include members of the phyla Firmicutes, Bacteroidetes, Proteobacteria, and Actinobacteria. Genera such as *Streptococcus*, *Prevotella*, *Veillonella*, and *Haemophilus* are frequently detected, often derived from the oral cavity via microaspiration (Li R. et al., 2024; Natalini et al., 2023; Huffnagle et al., 2017). This equilibrium, however, is disrupted in disease states. In conditions such as asthma, COPD, pneumonia, and acute respiratory distress syndrome (ARDS), lung microbial communities undergo significant shifts in composition and function (Li R. et al., 2024). For instance, exacerbations of COPD are characterized by increased abundance of *Haemophilus*, *Moraxella*, *Klebsiella*, and *Pseudomonas*, accompanied by elevated levels of proinflammatory cytokines like TNF-*α*. These microbial shifts not only reflect but actively contribute to disease pathogenesis by modulating immune responses and enhancing tissue inflammation (Huffnagle et al., 2017; Xue et al., 2023; Belizário et al., 2023). Importantly, lung microbiota also interacts with systemic immunity. Dysbiosis may skew T cell differentiation toward inflammatory subsets such as Th1 and Th17, which in turn perpetuate pulmonary inflammation. Conversely, the presence of immunomodulatory genera like *Lactobacillus* and *Veillonella* in certain COPD phenotypes suggests a potential for microbiota-based immune regulation (Xue et al., 2023).

The lung microbiome also plays a role in cancer development and progression. Several studies have identified distinct microbial signatures associated with lung cancer. Enrichment of genera such as *Streptococcus*, *Veillonella*, *Megasphaera*, and *Acidovorax* has been linked to tumor tissues and may influence oncogenesis through chronic inflammation, immune evasion, or modulation of host signaling pathways (Natalini et al., 2023; Ramírez-Labrada et al., 2020; Gilbert et al., 2025). Furthermore, microbial dysbiosis has been associated with poorer responses to immunotherapy, and antibiotic use before ICBs treatment correlates with reduced progression-free and overall survival (Thapa et al., 2024; Zapata-García et al., 2024).

Recent evidence also supports the concept of a bidirectional gut-lung axis, wherein microbial metabolites and immune signals from the gut influence lung homeostasis and vice versa (Dora et al., 2024). For example, gut-derived SCFAs can reduce airway inflammation, while respiratory infections can perturb gut microbial communities (Natalini et al., 2023; Levan et al., 2019). Despite these advances, challenges remain in defining a “healthy” lung microbiome and translating observational findings into effective clinical interventions. Contamination during sampling, low microbial biomass, and interindividual variability complicate analysis. Nevertheless, the therapeutic potential of manipulating the lung microbiota through probiotics, targeted antimicrobials, or even microbial transplantation represents a promising frontier in respiratory medicine (Natalini et al., 2023; Gilbert et al., 2025; Belizário et al., 2023).

### Skin microbiome

The skin microbiome is a rich and dynamic ecosystem composed of diverse microorganisms, including bacteria, fungi, viruses, and mites that reside on the skin’s surface and within its appendages (Chen et al., 2022; Figure 1). This microbiota plays a fundamental role in maintaining cutaneous health, educating the immune system, and preventing colonization by pathogenic organisms (Bautista et al., 2025a). Human skin, with its highly heterogeneous structure, featuring sebaceous, moist, and dry regions, provides distinct ecological niches that support site-specific microbial communities (Byrd et al., 2018; Grice and Segre, 2011). The most dominant bacterial phyla on the skin are *Actinobacteria*, *Firmicutes*, *Proteobacteria*, and *Bacteroidetes*. These include species such as *Cutibacterium acnes*, *Staphylococcus epidermidis*, and *Corynebacterium* spp., which serve critical roles in maintaining skin homeostasis. These commensals not only provide colonization resistance but also produce antimicrobial peptides (AMPs), modulate immune responses, and degrade skin lipids for nutrient acquisition (Byrd et al., 2018; Grice and Segre, 2011; Nakatsuji et al., 2021a; Wu and Xie, 2025). For instance, coagulase-negative staphylococci (CoNS) can secrete bacteriocins and autoinducing peptides that suppress the growth and virulence of *Staphylococcus aureus*, a common skin pathogen that frequently exacerbates atopic dermatitis (AD) (Nakatsuji et al., 2021a, 2021b).

Advanced sequencing technologies, including 16S rRNA gene sequencing and shotgun metagenomics, have revealed an extraordinary taxonomic and functional diversity within the skin microbiome (Wensel et al., 2022). Unlike culture-based methods that are biased toward easily cultivable species, these techniques allow for strain-level resolution and functional annotation. Such resolution is crucial, as strains within the same species (e.g., *S. epidermidis*) may possess dramatically different immunological and antimicrobial properties (Byrd et al., 2018; Grice and Segre, 2011).

The skin microbiome is also closely intertwined with immune regulation. Microbial signals can influence both innate and adaptive immunity through Toll-like receptors (TLRs), cytokine cascades, and AMP induction (Liu et al., 2023). For example, *Cutibacterium acnes* can promote inflammation via SCFA-mediated histone deacetylase inhibition in sebocytes, contributing to the pathogenesis of acne (Sanford et al., 2019). In contrast, specific microbial metabolites and cell-wall components can downregulate proinflammatory responses and promote tissue repair. *Staphylococcus hominis* and *Roseomonas mucosa* have demonstrated the ability to modulate epithelial responses and restore barrier function in AD, supporting the potential of bacteriotherapy for skin disorders (Nakatsuji et al., 2021b; Myles et al., 2020).

Disruptions in the skin microbiota are increasingly recognized as contributors to disease. In AD, for instance, overgrowth of *S. aureus* promotes inflammation, suppresses AMP expression, and correlates with disease severity (Di Domenico et al., 2019). Patients with AD often lack protective commensals such as CoNS that can inhibit *S. aureus* colonization through quorum sensing interference and bacteriocin production. Restoring these protective strains via topical application has shown efficacy in clinical trials, reducing microbial burden and improving symptoms (Nakatsuji et al., 2021b; Myles et al., 2020). Moreover, the skin microbiota can shape disease outcomes beyond classical dermatological conditions, as illustrated by its contribution to inflammation and delayed healing in cutaneous leishmaniasis through IL-1*β* signaling pathways (Farias Amorim et al., 2023).

Age is another important determinant of skin microbiome structure. In infants, the skin microbiome is seeded predominantly by maternal sources and evolves in response to environmental exposures, skin maturation, and immune development (Wang Y. R. et al., 2022). A recent genome catalog of early-life skin microbiota expanded our understanding of its diversity and revealed functional elements related to immune modulation and skin barrier support, such as sphingolipid biosynthesis and AMP-associated pathways (Shen et al., 2023). Lastly, the skin microbiome is not merely a passive inhabitant of the epidermis but an active participant in the maintenance of cutaneous and systemic health. Advances in multi-omic tools have uncovered their contributions to immune regulation, disease modulation, and therapeutic innovation (Farias Amorim et al., 2023; Nakatsuji et al., 2021b; Grice and Segre, 2011; Byrd et al., 2018; Gilbert et al., 2025).

### Vaginal microbiome

The human vaginal microbiome plays a pivotal role in maintaining reproductive and systemic health (Figure 1). It is a low-diversity but highly specialized ecosystem, typically dominated by *Lactobacillus* species such as *L. crispatus*, *L. iners*, *L. jensenii*, and *L. gasseri*, which collectively contribute to the production of lactic acid and the maintenance of an acidic pH (3.5–4.5) that inhibits pathogen colonization (Pramanick et al., 2019; Kwon and Lee, 2022; Lebeer et al., 2023; Spencer et al., 2023). These bacteria also interact with host epithelial cells, modulate local immune responses, and may be vertically transmitted to offspring during birth, potentially influencing neonatal immune development and long-term health outcomes (McCauley et al., 2022; Gilbert et al., 2025). The vaginal microbiota is classified into community state types (CSTs), with four dominated by *Lactobacillus* species (CST-I: *L. crispatus*; CST-II: *L. gasseri*; CST-III: *L. iners*; CST-V: *L. jensenii*) and CST-IV characterized by high bacterial diversity, often including *Gardnerella*, *Atopobium*, *Prevotella*, and other anaerobes (Kwon and Lee, 2022; Lebeer et al., 2023). Notably, *L. iners*, while common, is often associated with transitional or dysbiotic states and lacks the robust protective functions attributed to *L. crispatus* (Pramanick et al., 2019; Kwon and Lee, 2022). The microbial community shifts dynamically in response to hormonal changes, menstruation, childbirth, and lifestyle factors, as shown in large-scale cohorts such as the Isala project (Lebeer et al., 2023).

Dysbiosis of the vaginal microbiome is implicated in numerous clinical conditions. In bacterial vaginosis (BV), *Lactobacillus* abundance is depleted and replaced by polymicrobial communities rich in *Gardnerella vaginalis*, *Fannyhessea vaginae*, and *Prevotella* spp., contributing to biofilm formation, elevated pH, and chronic inflammation (Lebeer et al., 2023; Huang et al., 2024). Although often asymptomatic, BV increases the risk of preterm birth, pelvic inflammatory disease, and susceptibility to sexually transmitted infections (Pramanick et al., 2019; Kwon and Lee, 2022; Lev-Sagie et al., 2019). In contrast, vulvovaginal candidiasis (VVC), caused primarily by *Candida albicans*, reflects a fungal overgrowth in an otherwise *Lactobacillus*-rich environment. A recent study revealed that VVC-associated *C. albicans* strains induce stronger epithelial cell detachment and reduced type I interferon responses, distinguishing them from commensal strains and suggesting differential virulence potential (Reid et al., 2001). These insights may support the development of phenotypic assays to better stratify VVC risk and refine antifungal therapy (Sala et al., 2023). Probiotic strategies using specific *Lactobacillus* strains such as *L. rhamnosus* GR-1 and *L. fermentum* RC-14 have demonstrated the ability to restore normal vaginal flora, particularly in women with recurrent BV or depleted *Lactobacillus* communities (Reid et al., 2001). These strains, when administered orally or vaginally, can reestablish microbial balance and reduce recurrence rates (Gilbert et al., 2025). However, efficacy may vary based on host factors, strain-specific properties, and baseline microbiota composition (McCauley et al., 2022).

Vaginal microbiota transplantation (VMT) has recently emerged as a promising intervention for refractory BV. In a proof-of-concept trial, VMT from screened healthy donors led to long-term remission in most patients with recurrent BV, with restoration of *Lactobacillus* dominance and symptomatic relief (Lev-Sagie et al., 2019). These results parallel the success of FMT in gastrointestinal conditions, yet highlight the need for regulatory oversight, donor standardization, and longitudinal safety data (Gilbert et al., 2025). The vaginal microbiome influences mucosal immunity through both direct and indirect mechanisms. *Lactobacillus* species, especially *L. crispatus*, can modulate host immunity via secretion of bioactive compounds, including β-carboline alkaloids that suppress type I interferon responses and promote immune tolerance (Gilbert et al., 2025; Reid et al., 2001). Moreover, vertically transmitted *Lactobacilli* such as *L. jensenii* have been shown to inhibit activation of antigen-presenting cells and attenuate allergic responses in animal models, suggesting a role in intergenerational immune imprinting (McCauley et al., 2022).

The recent construction of the Vaginal Microbial Genome Collection, encompassing over 33,000 reference genomes across bacteria, fungi, and viruses, has substantially expanded our understanding of vaginal microbial diversity and function (Huang et al., 2024). This database revealed that over 85% of viral operational taxonomic units and many bacterial species remain uncultured, underscoring the vast unexplored diversity of the vaginal ecosystem. Moreover, many functional genes linked to immune modulation, epithelial adhesion, and biofilm formation remain to be experimentally validated (Huang et al., 2024; Kwon and Lee, 2022). Beyond typical microbiota, opportunistic colonizers like Group B *Streptococcus* (GBS) exploit host-microbiome interactions for persistence in the vaginal niche. Recent work has shown that GBS uses a Type VII secretion system (T7SS) with subtype-specific effectors that can influence epithelial colonization and immune modulation (Spencer et al., 2023). These mechanisms may help explain the variability in GBS carriage and its implications for neonatal infection risk.

### Microbiome-based therapeutics and clinical translation

**Figure 2:**
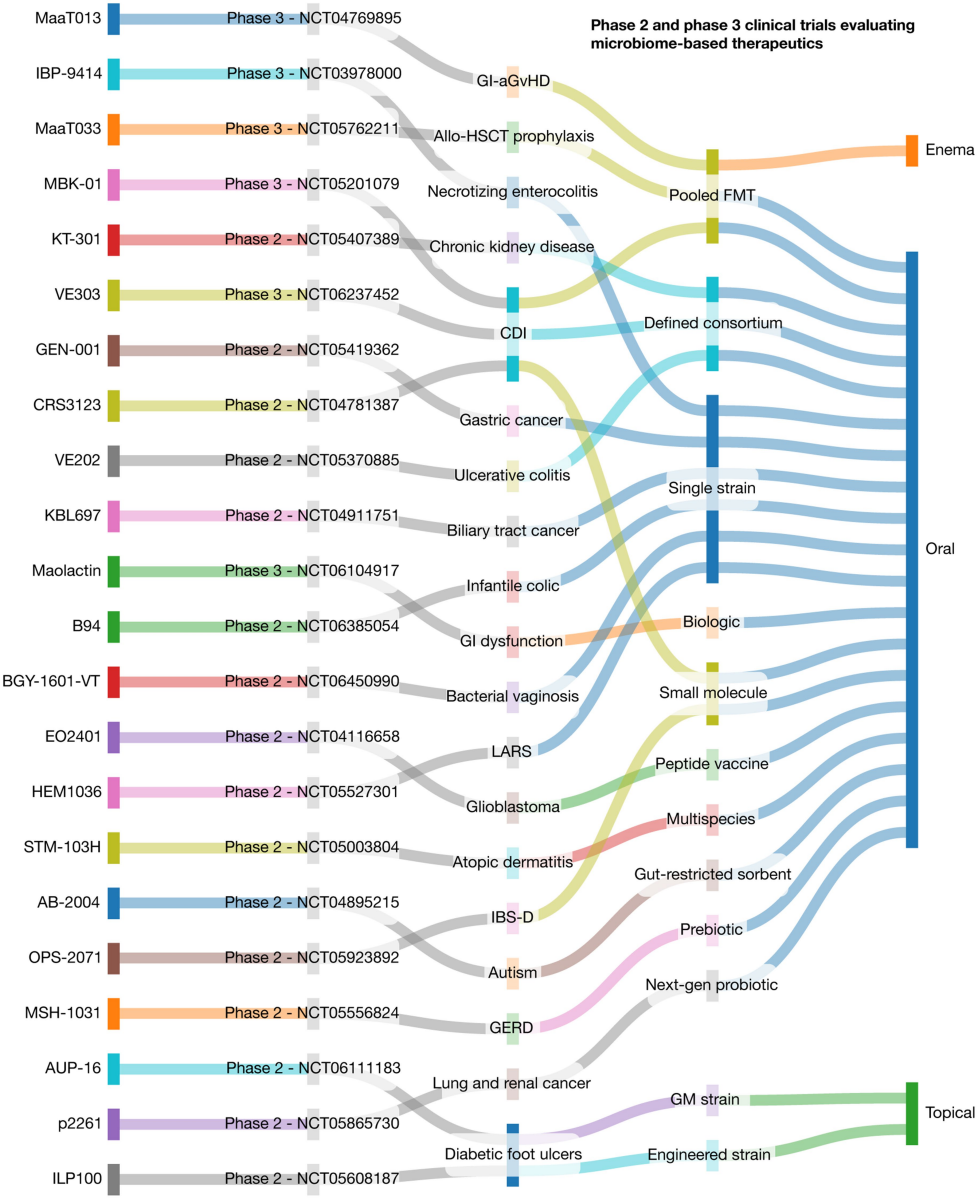
Landscape of phase 2 and 3 clinical trials evaluating microbiome-based therapeutics. This Sankey plot visualizes the translational flow of microbiome-based therapies across key domains: therapeutic agent, target disease, modality of intervention, delivery route, clinical trial phase and number. Each stream illustrates the relationship between components, emphasizing the diversity of clinical applications and development strategies within the microbiome field. Data include 22 active trials representing interventions in gastrointestinal, metabolic, neurologic, oncologic, dermatologic, and pediatric disorders. CDI: *Clostridioides difficile* infection; GI-aGvHD: Gastrointestinal acute graft-versus-host disease; Allo-HSCT: Allogeneic hematopoietic stem cell transplantation; GERD: Gastroesophageal reflux disease; IBS-D: Irritable bowel syndrome with diarrhea; LARS: Low anterior resection syndrome; Pooled FMT: Pooled fecal microbiota transplant; GM strain: Genetically modified microbial strain. This was designed through the SankeyMATIC code available at github.com/nowthis/sankeymatic.

Microbiome-based therapeutics have evolved from empirical FMT to rationally engineered interventions, including defined microbial consortia, genetically modified strains, prebiotics, and phage therapies (Mimee et al., 2016; Bajaj et al., 2022; Gulliver et al., 2022). As of 2025, at least 22 industry-sponsored Phase 2 and 3 trials (with NCT registration) are underway, targeting conditions across gastroenterology, oncology, neonatology, dermatology, and neurology (Figure 2). In *Clostridioides difficile* infection (CDI), MBK-01 (NCT05201079) and VE303 (NCT06237452) are leading Phase 3 candidates demonstrating efficacy and safety over fidaxomicin or donor-derived FMT (Reigadas et al., 2020). In neonatology, IBP-9414 (NCT03978000) is being tested to prevent necrotizing enterocolitis in preterm infants. MaaT Pharma’s MaaT013 (NCT04769895) and MaaT033 (NCT05762211) address gastrointestinal graft-versus-host disease using pooled FMTs. RDC Clinical’s Maolactin (NCT06104917) targets GI dysfunction, and Kibow Biotech’s KT-301 (NCT05407389) is under Phase 2 evaluation for chronic kidney disease (Gilbert et al., 2025).

In oncology, EO2401 (NCT04116658) is a microbial peptide vaccine for glioblastoma, while GEN-001 (NCT05419362) and KBL697 (NCT04911751) are being tested in combination with checkpoint inhibitors for gastric and biliary tract cancers (Reardon et al., 2023; Lee J. et al., 2024). Inflammatory and immune-related conditions are also key targets: VE202 (NCT05370885) and STM-103H (NCT05003804) are consortia being tested in ulcerative colitis and atopic dermatitis, respectively, and AUP-16 (NCT06111183) is a genetically modified topical strain for diabetic foot ulcers (Gilbert et al., 2025). Neurological and metabolic indications include AB-2004 (NCT04895215) for ASD, MSH-1031 (NCT05556824) for GERD, and B94 (NCT06385054) for infantile colic. Other novel applications include BGY-1601-VT for bacterial vaginosis (NCT06450990), p2261 for lung and renal cancers (NCT05865730), OPS-2071 for IBS-D (NCT05923892), and HEM1036 for low anterior resection syndrome (NCT05527301). Additionally, ILP100-Topical (NCT05608187) and CRS3123 (NCT04781387) are being tested for diabetic ulcers and CDI, respectively (Gilbert et al., 2025). These trials reflect a critical maturation of the field toward precision-targeted, regulated therapeutics—yet ongoing challenges such as interindividual microbial variability and the need for validated biomarkers must be addressed to enable broader clinical adoption.

### Risk and challenges in microbiome-based therapeutics

While microbiome-based therapies offer significant clinical promise, they are accompanied by a spectrum of risks and challenges that must be carefully considered to ensure safe and effective translation into clinical practice. One of the foremost concerns is the unpredictable ecological outcome of introducing live microbes, via FMT, probiotics, or engineered consortia, into a host’s existing microbial ecosystem. These interventions can inadvertently disrupt microbial balance, promote pathobiont overgrowth, or facilitate horizontal gene transfer, including the spread of antibiotic resistance genes (Gulliver et al., 2022; Hitch et al., 2022). Safety concerns are particularly acute in vulnerable populations, such as immunocompromised patients or those with epithelial barrier dysfunction. Cases of sepsis and transmission of multidrug-resistant organisms have been reported in association with improperly screened FMT, underscoring the need for rigorous donor selection and microbial quality control (Matuchansky, 2015). Even probiotics, traditionally perceived as benign, have in rare cases led to bloodstream infections or immune complications, especially when administered outside of tightly controlled clinical settings (Sarita et al., 2024).

One of the foremost challenges is interindividual variability in microbiome composition and function. Differences in baseline ecology can alter colonization success, metabolite production, and immune modulation, leading to divergent clinical outcomes for the same intervention. For example, probiotics shown to be effective in one cohort may fail in another due to differences in dietary patterns, antibiotic history, or host genetics (Murga-Garrido et al., 2021; Rothschild et al., 2018; Qin et al., 2022). Longitudinal studies also reveal that microbiome communities are highly individualized and remain stable over years, highlighting the difficulty of standardizing interventions across populations (Zhou X. et al., 2024). This underscores the need for predictive microbial and host biomarkers to guide therapeutic personalization, while also recognizing that technical variability from sequencing platforms, DNA extraction methods, and bioinformatics pipelines can further produce inconsistent taxonomic and functional profiles (Gulyás et al., 2024). To mitigate these discrepancies, benchmarking initiatives, standardized mock communities, and spike-in controls have been developed to provide internal reference points for bias correction (Bokulich et al., 2020; Tourlousse et al., 2022). More recently, AI-driven bioinformatic frameworks and cross-platform normalization strategies have emerged as powerful tools for harmonizing heterogeneous datasets and enhancing reproducibility across multi-cohort studies.

To ensure reproducibility across microbiome studies, standardized protocols must be implemented at every stage of the workflow, including sample collection, processing, and data analysis. At the pre-analytical level, harmonization of collection methods, stabilization agents, and storage conditions is essential to minimize variability introduced by environmental and handling factors. During DNA extraction and library preparation, the use of validated kits, bead-beating settings, and standardized primers should be consistently reported, alongside the incorporation of mock communities and spike-in controls to benchmark technical bias (Bokulich et al., 2020; Tourlousse et al., 2022). Sequencing protocols should define minimum depth requirements, quality thresholds, and control samples across runs to enable cross-study comparability. Equally critical are bioinformatic standards: version-locked pipelines, consistent reference databases, and compositional data–aware statistical frameworks that prevent false discoveries (Gulyás et al., 2024; Rosati et al., 2024). Finally, adherence to community reporting guidelines and transparent deposition of raw data, metadata, and analysis workflows in public repositories will provide the foundation for reproducibility and equity in clinical translation (Tedersoo et al., 2021).

From a regulatory standpoint, microbiome-based therapeutics, particularly those involving live or genetically modified organisms, face significant challenges in classification, approval, and commercialization. Regulatory frameworks vary across regions, and there is ongoing debate over how to best ensure safety, consistency, and efficacy in products that cannot be defined by a single molecular entity. The lack of harmonized standards for manufacturing, donor screening, and microbiome characterization further complicates product development and comparability across clinical trials (Guglielmetti et al., 2025). Finally, the long-term consequences of microbial manipulation remain largely unknown. While short-term safety data are accumulating, few studies have examined the durable effects of altering microbiome composition during critical developmental windows or in chronic diseases. Given the deep integration of the microbiome into host metabolic, immune, and neurological systems, unintended outcomes may emerge only over extended follow-up (Cox et al., 2014; Quigley and Gajula, 2020).

In summary, a balanced view of microbiome-based therapeutics must weigh their clinical potential against documented and theoretical risks. Addressing safety, interindividual variability, technical heterogeneity, regulatory gaps, and mechanistic uncertainties through standardized protocols, benchmarking efforts, mechanistic modeling, and robust trial design will be critical to ensure these interventions are both effective and safe across diverse patient populations (Metris et al., 2025). Although the majority of microbiome–disease studies remain associative, causal inference is increasingly supported by mechanistic approaches such as gnotobiotic transfer experiments, engineered microbial consortia, and metabolite add-back studies, which demonstrate that specific taxa and functions can directly modulate host immune and metabolic pathways (Sivan et al., 2015; Vétizou et al., 2015). Longitudinal human cohorts with dense multi-omic profiling, combined with statistical frameworks such as causal graphs and target trial emulation, are beginning to address temporality and reduce confounding (Lloyd-Price et al., 2019; Hernán and Robins, 2016). Complementary strategies, including N-of-1 trials, synthetic communities, Mendelian randomization using host genetic variants, and stable-isotope tracing to link microbial metabolites to host physiology, provide rigorous tools to move from correlation to causation (Zhou D. et al., 2024). Moving forward, reproducibility will depend on harmonized protocols, mock-community benchmarking, and inclusive, multi-site longitudinal studies that ensure findings generalize across diverse populations (Sinha et al., 2017; Pasolli et al., 2019).

### Conclusions and future perspectives

The clinical translation of microbiome research is no longer a distant prospect but an emerging reality that is reshaping diagnostics, therapeutics, and personalized medicine (Gilbert et al., 2025). Across diverse anatomical sites such as the gut, vagina, lung, skin, and oral cavity, microbial communities orchestrate a myriad of physiological functions, from immune calibration and metabolic regulation to neuroendocrine signaling and barrier integrity (Ma et al., 2024). The insights derived from advanced multi-omics approaches, mechanistic modeling, and interventional trials have uncovered fundamental principles underpinning host–microbe symbiosis and dysbiosis (Macpherson et al., 2023; Natalini et al., 2023; Huang et al., 2024).

Therapeutic modulation of the microbiome has shown promising results across a range of clinical settings. In metabolic syndrome, for example, interventions such as intermittent fasting have demonstrated the capacity to reduce blood pressure and body weight while reshaping gut microbial composition toward SCFA-producing taxa with anti-inflammatory properties (Maifeld et al., 2021). Inflammatory bowel disease has become a prototype condition for microbiome-targeted therapy, with phage consortia successfully suppressing pathobionts like *Klebsiella pneumoniae* in murine models, offering a viable alternative to broad-spectrum antibiotics (Federici et al., 2022). Similarly, in neonates, bioactive components in breast milk such as complement proteins, modulate microbial colonization and confer protection against enteric infections through a C1-dependent mechanism, underscoring the role of maternal–microbiota–immune crosstalk in early life programming (Xu et al., 2024).

Emerging research demonstrates that microbial signals extend beyond local niches, influencing systemic physiology through inter-organ communication networks. The gut–brain–immune axis integrates microbial metabolites and neuroactive molecules that regulate microglial maturation, blood–brain barrier permeability, and behavior (Macpherson et al., 2023; O’Riordan et al., 2025). The lung microbiome, once considered negligible, is now recognized as a regulator of respiratory immune tone, with dysbiosis contributing to asthma, COPD, and altered immunotherapy response in lung cancer (Natalini et al., 2023). Likewise, dysbiosis in the oral and skin microbiomes has been linked to systemic inflammation, impaired wound healing, and autoimmune predisposition (Kerns et al., 2023; Byrd et al., 2018).

Despite these advances, major challenges persist. The definition of a “healthy” microbiome remains elusive due to interindividual variability driven by geography, age, diet, lifestyle, and host genetics (Lloyd-Price et al., 2016; Human Microbiome Project Consortium, 2012). Predictive biomarkers for treatment success are still underdeveloped, and therapeutic outcomes remain heterogeneous. Furthermore, large proportions of microbial “dark matter” remain uncultured and functionally uncharacterized (Huang et al., 2024). The integration of microbial diagnostics into clinical workflows is further complicated by regulatory uncertainty, safety considerations, and the need for longitudinal data in diverse populations (Gilbert et al., 2025). A major obstacle in advancing precision medicine is the integration of microbiome data with other omics layers, including genomics, transcriptomics, metabolomics, and epigenomics. Unlike host-derived molecular data, microbiome datasets are inherently sparse, compositional, and noisy, which complicates statistical modeling and alignment with continuous data types (Busato et al., 2023). Technical variability introduced by differences in sampling, sequencing platforms, and bioinformatic pipelines leads to pronounced batch effects, further limiting comparability across studies and cohorts (Yu et al., 2024). In addition, a large fraction of microbial genes and metabolites remains functionally uncharacterized, restricting the capacity to link microbial features with host pathways in a biologically meaningful manner. These challenges are compounded by the high temporal and spatial dynamics of microbial communities, which introduce variability absent in relatively stable host genomes. As a result, causal inference across multi-omic layers remains difficult, and reproducibility is often limited (Mangnier et al., 2025). Addressing these obstacles will require the adoption of advanced computational frameworks, including bioinformatics pipelines and AI-driven integration tools, capable of harmonizing heterogeneous datasets while ensuring interpretability for clinical application (Mani et al., 2025).

Future strategies must explicitly address variability by leveraging personalized stratification frameworks, focusing on functional rather than taxonomic markers, integrating microbiome data with host multi-omic layers, and ensuring inclusivity in cohort design to enhance generalizability (Schupack et al., 2022; Muller et al., 2024; Andreu-Sánchez et al., 2025).

Looking ahead, clinical microbiome research must prioritize the development of mechanistic models that can explain and predict host–microbiota interactions across tissues and disease states (Gibbons et al., 2022). Standardization of microbial reference genomes, expansion of multi-kingdom and strain-level annotations, and implementation of high-throughput functional assays will be essential to identify and validate therapeutic targets (Huang et al., 2024). Personalized medicine approaches should leverage microbial metrics (taxonomic composition, metabolite profiles), immune response signatures, epigenetic signatures, and circadian rhythm profiles to stratify patients and tailor interventions accordingly (Pérez-Villa et al., 2023; Bautista et al., 2025b; Ocaña-Paredes et al., 2024; López-Cortés et al., 2021).

To operationalize these goals, computational tools and machine learning algorithms are indispensable. Multi-omics integration frameworks such as MOFA+ and DIABLO enable the extraction of shared biological signals across microbial, metabolic, immune, and host genomic layers (Argelaguet et al., 2020; Singh et al., 2019). Deep learning architectures, including variational autoencoders and graph-based models, allow for biologically constrained feature learning from highly dimensional data (Baig et al., 2023; Kim et al., 2019). These approaches support patient stratification into molecular endotypes, prediction of therapy response or toxicity using interpretable models such as elastic-net regression, gradient boosting, and survival forests, and the identification of actionable microbial or metabolic pathways through explainability techniques like SHAP values (Wang C. et al., 2022; Rynazal et al., 2023). Critically, computational pipelines must incorporate external validation, decision-curve analysis, and fairness checks to ensure clinical robustness and equitable deployment.

Interdisciplinary collaboration across microbiology, immunology, nutrition, neuroscience, and computational biology will be critical to address these complexities and maximize translational impact. Lastly, the human microbiome has moved from associative observation to actionable science. By decoding the molecular grammar through which microbial communities influence health and disease, we are now positioned to design microbiome-informed therapies that are precise, effective, and scalable. With careful attention to mechanistic rigor, safety, and interindividual variability, microbiome research is poised to reshape preventive and therapeutic paradigms across medicine (Macpherson et al., 2023; Gilbert et al., 2025; Huang et al., 2024; Natalini et al., 2023; Maifeld et al., 2021).

An important but often overlooked challenge in clinical microbiome research is health equity (Ma et al., 2024; Foxx et al., 2021). Current reference databases and classification tools are disproportionately derived from cohorts in North America, Europe, and East Asia, with limited representation from low- and middle-income countries. One example is the microbiome signature of The Cancer Genome Atlas (TCGA), in which more than 70% of participants are White (Spratt et al., 2016; Guerrero et al., 2018; Chen K. P. et al., 2023). This geographic bias not only restricts our understanding of global microbial diversity but also risks misclassification and reduced diagnostic accuracy when applying these tools across diverse populations (Blake, 2024). Addressing this imbalance requires large-scale initiatives that prioritize inclusivity, expand genome catalogs from underrepresented regions, and ensure equitable access to microbiome-informed therapies. Without deliberate attention to these gaps, the promise of microbiome-based precision medicine may inadvertently exacerbate global health disparities rather than reduce them (Fatumo et al., 2022; Pasolli et al., 2019; Lee S. et al., 2024).
